# Poststernotomy Osteomyelitis Presenting with Severe Sepsis and Rhabdomyolysis

**DOI:** 10.1155/2016/4507012

**Published:** 2016-04-10

**Authors:** Eugene M. Tan, Melissa Lyle, Kelly Cawcutt, Zelalem Temesgen

**Affiliations:** ^1^Mayo Clinic, 200 First Street SW, Rochester, MN 55905, USA; ^2^University of Nebraska Medical Center, S. 42nd Street and Emile Street, Omaha, NE 68198, USA

## Abstract

A 39-year-old male, who recently underwent a composite valve graft of the aortic root and ascending aorta for bicuspid aortic valve and aortic root aneurysm, was hospitalized for severe sepsis, rhabdomyolysis (creatine kinase 29000 U/L), and severe liver dysfunction (AST > 7000 U/L, ALT 4228 U/L, and INR > 10). Cardiac magnetic resonance imaging (MRI) findings were consistent with sternal osteomyelitis with a 1.5 cm abscess at the inferior sternotomy margin, which was contiguous with pericardial thickening. Aspiration and culture of this abscess did not yield any organisms, so he was treated with vancomycin and cefepime empirically for 4 weeks. Because this patient was improving clinically on antibiotics and did not show external signs of wound infection, there was no compelling indication for sternectomy. This patient's unusual presentation with osteomyelitis and rhabdomyolysis has never been reported and is crucial for clinicians to recognize in order to prevent delays in diagnosis.

## 1. Introduction

Poststernotomy osteomyelitis is a complication of open heart surgery in 1–5% of patients [1] that typically presents as purulent sternocutaneous fistulas and is commonly treated with combined medical and surgical therapy, including antibiotics, partial or total sternectomy, excision of infected costal cartilage, and chest reconstruction using muscle flaps to fill the chest gap [2]. The case herein illustrates a very unusual presentation that was treated nonsurgically.

## 2. Case Presentation

A 39-year-old male with a bicuspid aortic valve and aortic root aneurysm underwent a composite valve graft of the aortic root and ascending aorta. Surgery was indicated secondary to symptomatic presentation with exertional dyspnea and fatigue. He also had chronic systolic heart failure due to alcoholic cardiomyopathy, with an ejection fraction of 33%. In addition to alcohol, he had a history of polysubstance abuse including methamphetamine and tobacco. Other medical history was significant for Crohn's disease, which was in remission with weekly adalimumab injections. His immediate postoperative course was uneventful, and he was discharged on postoperative day 5 (POD#5) after adequate pain control was achieved.

On POD#6, he was rehospitalized for fever, night sweats, and syncope and was diagnosed with healthcare-associated pneumonia, which was treated with 8 days of levofloxacin from POD#6 to POD#14. During this readmission, he also developed a bloodstream infection secondary to* Staphylococcus epidermidis*, treated with 2 weeks of vancomycin from POD#6 to POD#20. He was discharged but returned on POD#23 to the Emergency Department with persistent fever, fatigue, confusion, dyspnea, cough, and abdominal pain for 10 days and met clinical criteria for severe sepsis (tachycardia of 134 beats per minute, tachypnea with respiratory rate of 32 breaths per minute, and elevated lactate of 3.0 mmol/L). Initial laboratory results also demonstrated an AST > 7000 U/L, ALT 4228 U/L, INR > 10, total bilirubin 1.4 mg/dL, direct bilirubin 0.7 mg/dL, creatine kinase 29000 U/L, and urine myoglobin 1235 mcg/L. He was taking warfarin at his prescribed dose of 3 mg daily with a target INR of 2.5–3.5. A viral hepatitis, acetaminophen, and urine drug screen were negative; there was no history of syncope or hypotension that would suggest ischemic hepatitis. He was empirically started on vancomycin, cefepime, and levofloxacin for sepsis of unknown origin.

**Figure 1:**
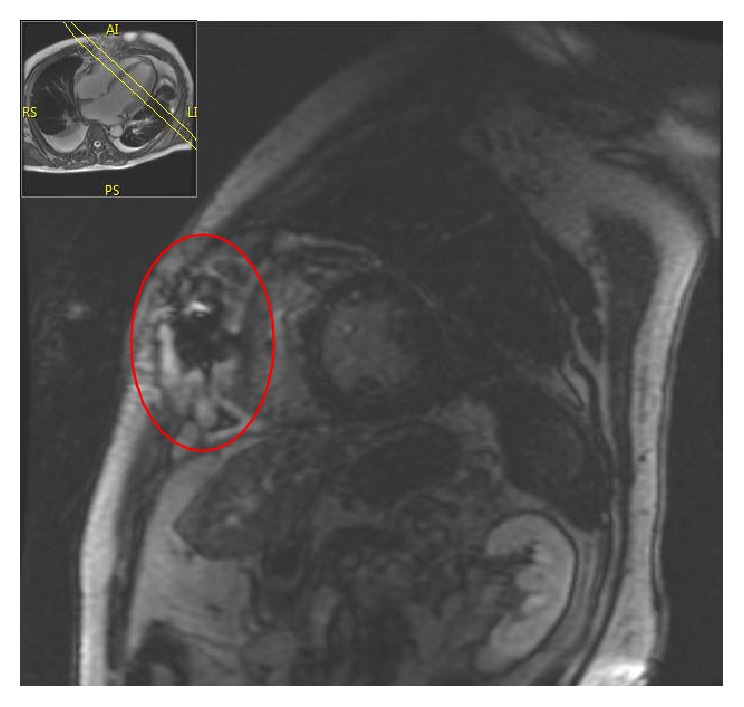
Cardiac MRI (short axis view) demonstrates findings consistent with sternal osteomyelitis, as evidenced by abnormal pericardial thickening and enhancement contiguous with a 1.5 cm fluid collection at the inferior margin of the sternotomy (red oval). Inset at top left illustrates plane of cross-sectional image (AI = anteroinferior, LI = left inferior, RS = right superior, and PS = posterosuperior).

**Figure 2:**
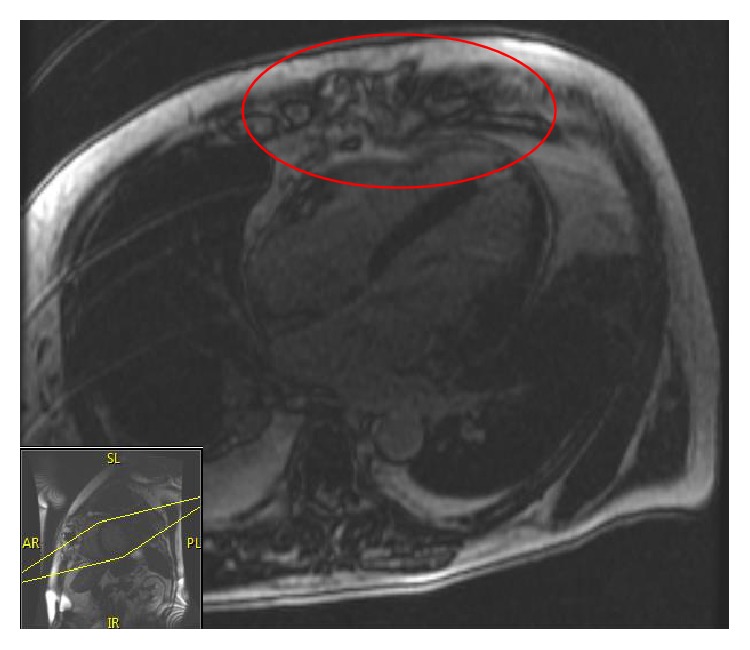
Cardiac MRI (four-chamber view) demonstrates a 1.5 cm small abscess contiguous with pericardial enhancement (red oval). Inset at bottom left demonstrates plane of cross-sectional image (SL = superolateral, PL = posterolateral, AR = anterior right, and IR = inferior right).

During this hospitalization, extensive evaluation with transesophageal echocardiogram, blood and urine cultures, and computed tomography (CT) of the head and abdomen showed no source of infection. The patient had no history of alcohol-related chronic liver disease, and the CT showed a normal-sized liver with no parenchymal enhancement or periportal edema. However, the cut margin of the distal sternotomy site was not well-defined and offset on CT of the chest appeared. Cardiac magnetic resonance imaging (MRI) findings were consistent with sternal osteomyelitis with a 1.5 cm abscess at the inferior sternotomy margin (Figure 1), which was contiguous with pericardial thickening (Figure 2). The patient underwent a CT-guided biopsy of the sternum on POD#28. No organisms were isolated, and cytology was nondiagnostic. Unfortunately, a pathologic analysis was not performed. Aspiration of the retrosternal fluid collection was attempted but did not yield any fluid. He had been treated with antibiotics for 6 days before the aspiration. Surgical intervention was determined to be unnecessary, as there were no external signs of sternal wound infection, and the patient showed rapid clinical improvement.

The laboratory abnormalities noted on admission were compatible with rhabdomyolysis. Evaluation for potential causes of rhabdomyolysis, including history of muscle injury, recent illicit drug use, myopathy, or viral etiologies, such as influenza, adenovirus, and herpes simplex, was negative. The patient's antibiotic therapy was deescalated to vancomycin and cefepime on POD#29. He continued to improve clinically; liver and muscle enzymes slowly decreased over 2 weeks. After administration of 2.5 mg of oral vitamin K and temporary discontinuation of warfarin, his INR returned to the therapeutic range (3.4) within three days after admission and was presumed due to a sepsis-related coagulopathy. He was discharged on POD#33. A follow-up MRI of the chest done 2 weeks later on POD#47 showed nonspecific thickening and enhancement of sternal soft tissue, but no fluid collection or abscess. The patient completed a total of 4 weeks of intravenous antibiotics with complete resolution of his infection and rhabdomyolysis.

## 3. Discussion

This patient's presentation with rhabdomyolysis was unusual for poststernotomy osteomyelitis and created a delay in diagnosis and management. The incidence of osteomyelitis presenting with rhabdomyolysis is unknown, as there are no reports in the literature describing this presentation. It is possible that the patient's osteomyelitis led to sepsis, which then caused rhabdomyolysis via muscle ischemia or cytokine-mediated muscle toxicity. Additional risk factors include chronic alcoholism, statin intake, hypokalemia, hypernatremia, and hypophosphatemia [3]. This patient did have a prior history of alcoholism but had been abstinent for over 1 year. He was not on a statin and did not have any of the aforementioned electrolyte abnormalities, nor any evidence of other predisposing factors for rhabdomyolysis: trauma, hypoxia, hyperthermia, hypothermia, endocrinologic or rheumatologic disorders, or inherited disorders of metabolism (e.g., McArdle disease) [4].

It is not known whether a particular pathogen is more likely to cause rhabdomyolysis. In a retrospective cohort study of 103 patients with community-acquired bacterial sepsis complicated by rhabdomyolysis, the predominant pathogens involved were gram-negative, namely,* Pseudomonas aeruginosa*,* Escherichia coli*, and* Klebsiella pneumoniae*. The most common focus of infection was the lung, but the exact type of lung infection (pneumonia, lung abscess, or empyema) was not specified [3]. In contrast, a separate study showed that bacterial sepsis-induced rhabdomyolysis was mostly associated with gram-positive pathogens, especially* Staphylococcus aureus* and* Enterococcus faecalis*, thus highlighting the need for further investigation on the typical pathogens associated with rhabdomyolysis [5].

Ultimately, as this patient was improving clinically on antibiotics and did not show external signs of wound infection, there was no compelling indication for sternectomy despite his presentation with sepsis and rhabdomyolysis. After completing 4 weeks of intravenous antibiotics, the patient was monitored for recurrence of infection as an outpatient, and he has continued to do well 14 months later. This case highlights the need for increased clinician awareness of the differential diagnosis of rhabdomyolysis, aggressive testing, and imaging if no clear cause is rapidly identified. Furthermore, this case emphasizes the need for future research on the relationship between rhabdomyolysis and infection.

Fortunately for this patient, once diagnosed, this patient's osteomyelitis was treated successfully, leading to a favorable outcome.
